# The Role of Bone Marrow Mesenchymal Stem Cells in the Treatment of Acute Liver Failure

**DOI:** 10.1155/2013/251846

**Published:** 2013-11-10

**Authors:** Shufang Yuan, Tao Jiang, Lihua Sun, Rongjiong Zheng, Nizam Ahat, Yuexin Zhang

**Affiliations:** ^1^Department of Infectious Diseases, First Affiliated Hospital of Xinjiang Medical University, Xinjiang 830011, China; ^2^State Key Laboratory Incubation Base of Xinjiang Major Diseases Research, The First Affiliated Hospital of Xinjiang Medical University, Urumqi, Xinjiang 830054, China; ^3^Key Laboratory of Xinjiang Medical Animal Model Research, Xinjiang 830011, China; ^4^Xinjiang University, Urumqi, Xinjiang 830011, China

## Abstract

*Objective.* This study is to investigate the effects of bone marrow mesenchymal stem cell (BMSC) transplantation on acute liver failure (ALF). *Methods.* BMSCs were separated from rat bone marrow, cultured, and identified by flow cytometry. Rat model with ALF was established by injecting D-galactosamine and lipopolysaccharide. Rats were randomly divided into the control group and BMSC transplantation group. The serum levels of alanine aminotransferase (ALT) and aspartate aminotransferase (AST) were measured at 24 h, 120 h, and 168 h after BMSC transplantation. Apoptosis was detected by TUNEL assay. The expression of VEGF and AFP proteins was detected by immunofluorescence. Caspase-1 and IL-18 proteins and mRNA were detected by immunohistochemistry and RT-PCR. *Results.* Compared with the control group, levels of ALT, AST, caspase-1 and IL-18 proteins, and mRNA in the transplantation group were significantly lower at 120 h and 168 h after BMSCs transplantation. Apoptosis was inhibited by BMSCs transplantation. The VEGF protein levels were increased with the improvement of liver function, and the AFP protein levels were increased with the deterioration of the liver function after BMSCs transplantation. *Conclusions.* BMSCs transplantation can improve liver function and inhibit hepatocyte apoptosis as well as promote hepatocyte proliferation in rat model with ALF.

## 1. Introduction

Acute liver failure (ALF) is a type of severe liver cell damage caused by viruses, drugs, toxins, or alcohol. ALF leads to hepatic encephalopathy, hepatorenal syndrome, severe infection, multiple organ failure, and even deaths [1–3]. The treatments of ALF include drug therapy, artificial liver therapy, stem cell transplantation, and liver transplantation. The artificial liver therapy may reduce mortality, but the efficacy is limited. Liver transplantation is the most effective method for treating ALF, but its application is limited because of lack of donor, immune rejection, and uses of immunosuppressive agents. In recent years, stem cell is a new way for the treatment of various diseases. Stem cell transplantation is a simple, flexible, reusable, weak, and immunogenic treatment of ALF [4–6].

The occurrence of ALF involves various inflammatory factors and cytokines, and ALF pathogenesis is closely related to the liver cell apoptosis [7]. It is reported that liver cell apoptosis was regulated by cellular proteins such as the Fas/FasL family, Bcl-2 family, and the caspase family [8]. It is reported that interleukin-18 (IL-18) and other cytokines were closely related to liver damage [9–11].

In this study, a rat model was established to study the efficacy of transplantation of BMSCs in the treatment of ALF. By detecting changes of serum alanine aminotransferase (ALT) and aspartate aminotransferase (AST), liver function improvement was compared before and after transplantation of bone marrow mesenchymal stem cells (BMSCs). By detection of dynamic changes in caspase-1 and IL-18 expression and hepatic cell apoptosis, the roles of BMSC transplantation on caspase-1, IL-18, and hepatic cell apoptosis of ALF rat were studied. The expression of vascular endothelial growth factors (VEGFs) and AFP protein in liver tissue was detected to explore the effect of BMSC transplantation on liver cell proliferation and to further explore the mechanism of BMSC transplantation in the treatment of ALF.

## 2. Materials and Method

### 2.1. Isolation and Culture BMSCs from Rats

BMSCs were isolated by using the wall-sticking method. Heparin (50000 U) was injected intraperitoneally. After injection for 15 min, rats were sacrificed. Subsequently, whole bone marrow of the bilateral tibia was collected and cells from the whole bone marrow were seeded in 75 cm^2^ petri dish at a concentration of 1 × 10^9^ cells/mL. After culturing for 24 h, the medium was changed to remove nonadherent cells. For propagating, adherent cells at the confluence of 70%–80% were trypsinized using 0.25% (w/v) trypsin/EDTA (Invitrogen, Carlsbad, CA, USA) and were replated at a 1 : 3 dilution. The BMSCs at the 3rd passage were identified by flow cytometry analysis and cultured for future experiments.

### 2.2. Animal Model Establishment

Sixty healthy male Sprague-Dawley (SD) rats, weighing from 250 g to 300 g (SPF-class), were provided by the Animal Center of Centers for Disease Control in Xinjiang (license number SCXK Xin from 2003 to 0002). This study was ethically approved by the Medical Ethics Committee of Xinjiang Medical University, Xinjiang, China. All animal experiments were conducted in accordance with the Europe Community Council Directive (86/EEC) and were conducted according to the principles of the Declaration of Helsinki. D-Galactosamine (10%) and lipopolysaccharide (0.005%) were intraperitoneally injected into the rats to prepare the ALF model. The 60 rats were divided into the control group (30 rats) and the BMSC transplantation group (30 rats). The rat samples in both groups were determined at three time points (24 h, 120 h, and 168 h after transplantation, resp.), with samples from 10 rats being determined at each time point in each group. Saline was intraperitoneally injected into rats in the control group. 1.4 × 10^7^ cells/kg of BMSCs were injected through the tail vein in the BMSC transplantation group and samples were collected at 24 h, 120 h, and 168 h after transplantation, respectively. Blood was collected from the portal vein and centrifuged to harvest serum. Serum ALT and AST changes were determined using automatic biochemical analyzer (Unicel DXC 800, BECKMAN COULTER) at various time points. Fresh liver tissue was collected for further experiments.

### 2.3. Flow Cytometric Analysis

The 3rd generation of cells was taken and digested by 2.5 g/L trypsin to prepare 1 × 10^9^/mL single cell suspension. The FITC-labeled antimouse CD45, CD29, CD11 or, CD90 antibody (BioLegend Company, USA) was added and mixed, and the solution was incubated at room temperature for 15 minutes. After two washes with PBS, it was analyzed by flow cytometric analysis.

### 2.4. Hematoxylin-Eosin (HE) Staining

Rat liver tissue was embedded in paraffin and serially sectioned into slices with a thickness of 4 **μ**m. Three to five slices were taken for HE staining. Five low-power fields (×200) and high-power fields (×400) were randomly selected for each slice under light microscopy to observe liver pathology after BMSC transplantation.

### 2.5. Terminal Deoxynucleotidyl Transferase dUTP Nick End Labeling (TUNEL)

The TUNEL assay kit (Roche Applied Science, Sweden) was used to detect apoptosis. Paraffin sections were routinely deparaffinized, rehydrated, and then rinsed by PBS. After blocking endogenous peroxidase activity by methanol, permeability liquid (1 g/L TritonX-100 was dissolved in 0.1% sodium citrate), TUNEL reaction solution and Converter-POD were added. Each slice was stained by 3,3′-diaminobenzidine (DAB), and liver cell apoptosis was observed under microscopy. The primary antibody was replaced by phosphate buffer on the positive specimens in negative control. The brown particles in the nucleus were considered as apoptosis-positive cells. Five fields were randomly selected in each slice under high-power field (400x). The number of positive liver cells and the total number of liver cells in each field were counted to calculate the percentage of positive liver cells. Positive cell rate <5% in each slice was considered as (−), 5%–25% was considered as (+), 25%–50% was considered as (++), and more than 50% was considered as (+++).

### 2.6. Immunohistochemical Detection

The samples were rehydrated and treated with 3% H_2_O_2_. The caspase-1 antibody (cat#, ab17820, 1 : 100, Abcam, MA, USA) or IL-18 antibody (cat#, sc-6179, 1 : 100, Santa Cruz Biotechnology, CA, USA) was added and preserved at 4°C overnight. Biotin-labeled secondary antibody and streptavidin-biotin-peroxisome solution were added, and the samples were stained by DAB, restained by hematoxylin, and sealed with neutral gum. Samples treated with phosphate buffer served as the negative control. Cells stained brown were considered positive.

### 2.7. Immunofluorescence Detection

The samples were prepared according to the protocol. VEGF antibody (cat#, ab1316, 1 : 100, Abcam, Hong Kong; MA, USA) or the AFP antibody (cat#, sc-15375, 1 : 100, Santa Cruz Biotech, Inc., CA, USA) was used. FITC (cat#, MH14501415, Thermo Scientific, Pierce Biotechnology, Rockford, USA) labeled secondary antibody was added, and the solution was incubated for 15 minutes. Then 4′,6-diamidino-2-phenylindole (DAPI) was used to stain nucleus. The cells were observed under fluorescence microscopy. The numbers of fluorescent-labeled liver cells and the total numbers of liver cells in each field were counted.

### 2.8. RT-PCR

The TRIzol reagent kit (Invitrogen, Carlsbad, USA) was used to extract RNA of liver tissues. The cDNA synthesis kit was purchased from BBI Company, USA. Caspase-1, IL-18, and internal control primers were synthesized by Shanghai Sangon Biotechnology Company (Shanghai, China). The primer sequences are shown in Table 1. The StepOnePlus fluorescent quantitative analyzer (ABI, USA) was used to determine Ct value of product. The relative amount of 2^−ΔΔΔCt^ method was used to compare expression of gene caspase-1 and IL-18 in modeling and stem cell transplantation of different rat liver tissues.

### 2.9. Statistical Analysis

SPSS18.0 statistical software was used for statistical analysis. The data was expressed as mean ± SD. Group comparison was performed by analysis of variance with randomized block design. Count data was analyzed with variance after rank conversion. Correlation analyses were performed by the Pearson correlation analysis. A *P* value less than 0.05 was considered statistically significant. 

## 3. Results

### 3.1. Transplantation with BMSCs

**Figure 1:**
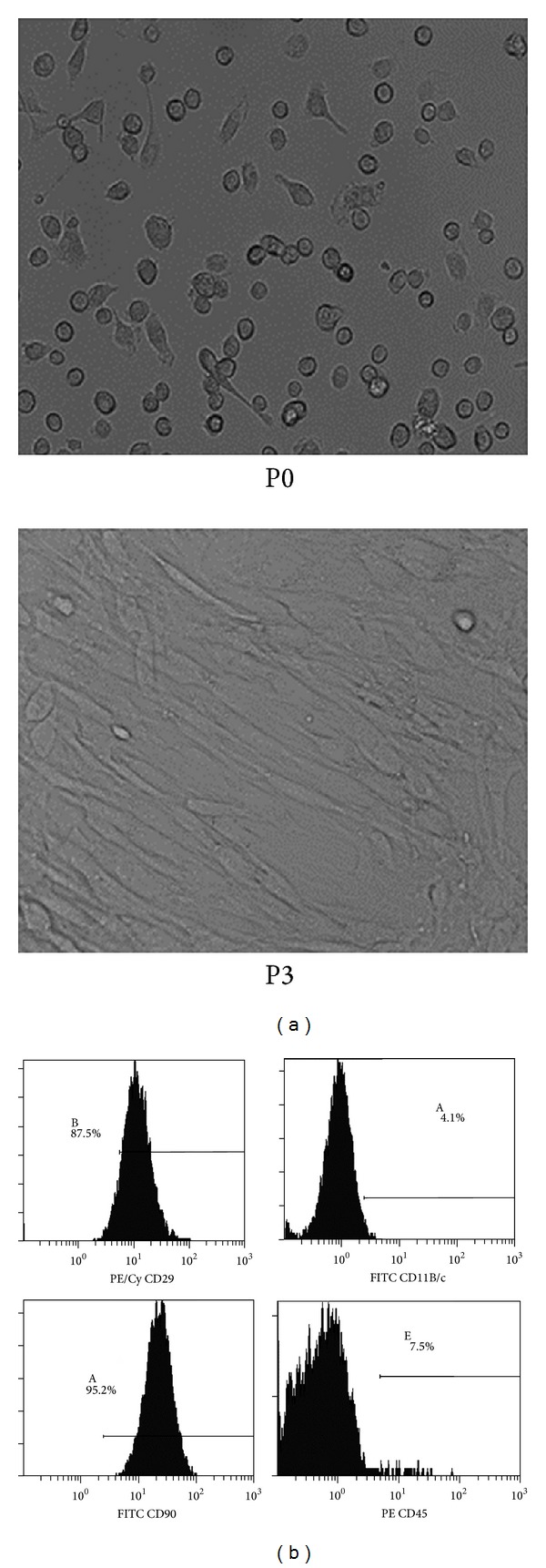
BMSCs prepared for the transplantation. (a) BMSCs in primary culture (P0, ×100) and the third generation (P3, ×100). (b) Determination of rat BMSCs phenotype by flow cytometry.

**Figure 2:**
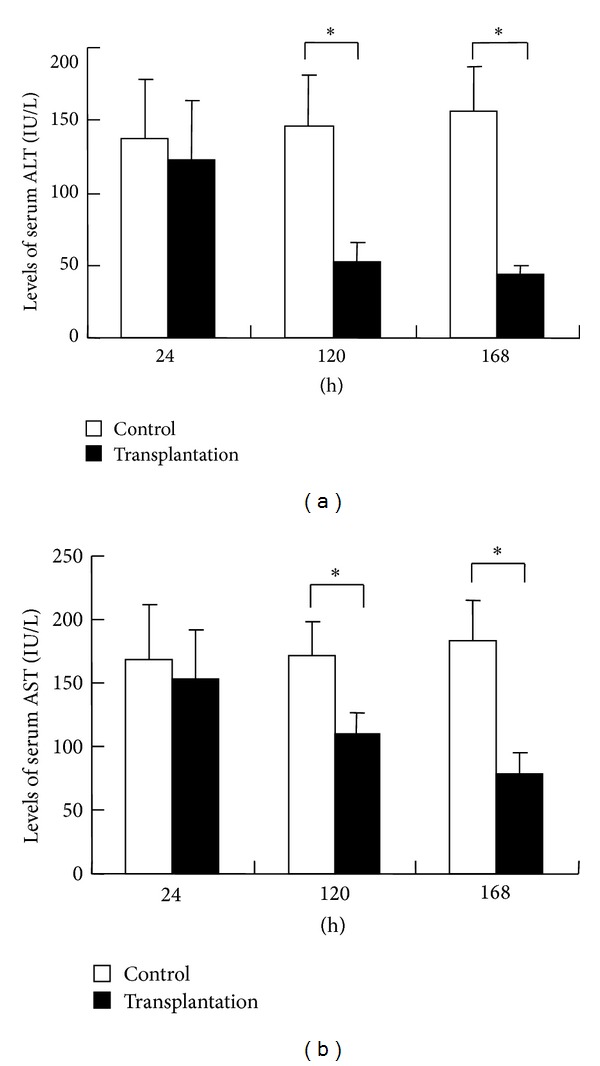
The levels of serum ALT and AST in both groups. At 24 h, 120 h, and 168 h after BMSCs transplantation, serum was collected from the control group and the transplantation group. (a) Levels of ALT in the serum detected by automatic biochemical analyzer. (b) Levels of AST in the serum detected by automatic biochemical analyzer; data were expressed as $\overset{-}{X}\pm S$. *P < 0.05, the control group versus the transplantation group.

To perform the transplantation, BMSCs were isolated (P0 generation, Figure 1(a)) and cultured until the third generation (P3 generation, Figure 1(a)). Flow cytometric analysis results (Figure 1(b)) indicated that BMSCs of P3 generation expressed MSC-specific markers CD29 (+) and CD90 (+), but not CD11 and CD45. It was found that the serum ALT and AST levels in the transplantation group were decreased when compared with those in the control group at 24 h, 120 h, and 168 h after transplantation (Figure 2). In each of the two groups, 10 rats were tested at each of the time points. The differences in the serum ALT and AST levels at 120 h and 168 h after transplantation between the two groups were statistically significant (*P* < 0.05) (Figure 2). These results suggest that BMSC transplantation can improve liver function in rats with ALF.

### 3.2. HE Staining of Liver Tissues of the Rats

**Figure 3:**
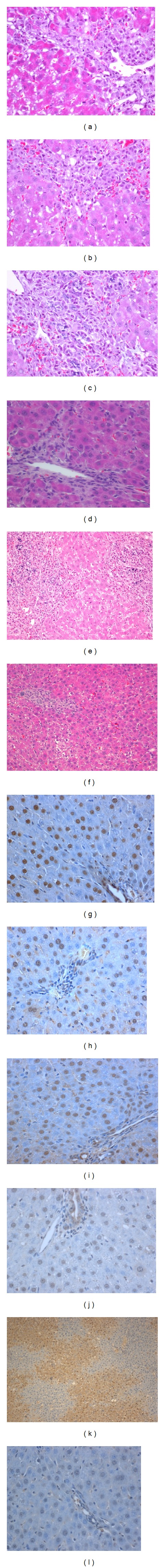
HE staining ((a)–(f)) and TUNEL assay ((g)–(l)) of the liver tissues. (a) Control group, 24 h (×400). (b) Transplantation group, 24 h (×400). (c) Control group, 120 h (×400). (d) Transplantation group, 120 h (×400). (e) Control group, 168 h (×200). (f) Transplantation group, 168 h (×200). (g) Control group, 24 h (×400). (h) Transplantation group, 24 h (×400). (i) Control group, 120 h (×400). (j) Transplantation group, 120 h (×400). (k) Control group, 168 h (×100). (l) Transplantation group, 168 h (×400).

To detect the possible changes induced by the transplantation in rat liver tissues, the liver tissues were harvested to perform HEstaining. HE staining results showed that the hepatic lobule lost their normal structure in the control group 24 h after the D-galactosamine injection. Liver cell degenerated and inflammatory cell infiltrated in portal area (Figure 3(a)). At 120 h and 168 h after modeling, liver cells in control group showed diffusive necrosis with extensive bridging necrosis, and inflammatory cell proliferated around periportal area (Figures 3(c) and 3(e)). At 120 h and 168 h after BMSCs transplantation, inflammatory cell infiltration in BMSCs transplantation group was significantly reduced (*P* < 0.05), and liver lobular was in recovery with bile duct hyperplasia in periportal area (Figures 3(d) and 3(f)). These results suggest that BMSCs could reduce inflammation response and necrosis in the liver.

### 3.3. BMSC Transplantation Reduces Apoptosis in the Liver Cells

To determine if the BMSC transplantation can reduce the ALF-related apoptosis, TUNEL was performed to examine the apoptosis levels in the rats of both groups. The results showed that in the control group, a lot of positively stained apoptotic cells of round shape and brown nucleus were observed (Figures 3(g), 3(i), and 3(k)). The numbers of the apoptotic cells increased in those at the 120 h (Figure 3(i)) and 168 h (Figure 3(k)) time points than in those at the 24 h time point (Figure 3(g)). In the BMSC transplantation group, the liver cell apoptosis at 24 h, 120 h, and 168 h after transplantation was detected (Figures 3(h), 3(j), and 3(l)). The apoptosis was significantly reduced when compared with that of the control group (*P* < 0.05) (Figure 3(i) versus Figure 3(j) and Figure 3(k) versus Figure 3(l)). These results suggest that BMSC transplantation can inhibit liver cell apoptosis. 

### 3.4. Expression of Caspase-1 and IL-18 Proteins

**Figure 4:**
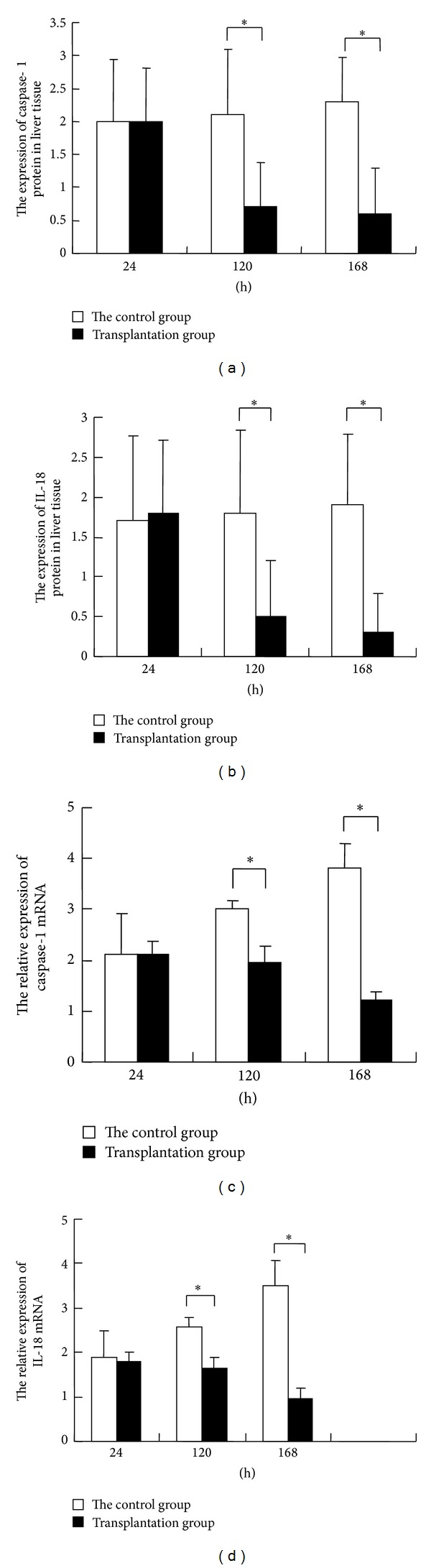
Expression of proteins related to changes induced by BMSC transplantation. ((a) and (b)) Immunohistochemical detection. The samples were rehydrated and treated with 3% H_2_O_2_. The caspase-1 antibody and IL-18 antibody were used. The ratios of the cell numbers were calculated. (a) Caspase-1 protein expression in liver tissue. (b) IL-18 protein expression in liver tissue. ((c) and (d)) RT-PCR detection of the mRNA expression in liver tissues. The StepOnePlus fluorescent quantitative analyzer was used to determine Ct value of product. The relative amount of 2^−ΔΔΔCt^ method was used to compare expression of gene caspase-1 and IL-18 in rat liver tissues. (c) Caspase-1 mRNA expression in liver tissue. (d) IL-18 mRNA expression in liver tissue.

Liver cell apoptosis is regulated by cellular proteins, such as various cytokines. The expression of caspase-1 and IL-18 proteins was determined by immunohistochemistry. The immunohistochemistry results showed that the protein expression of caspase-1 (Figure 4(a)) and IL-18 (Figure 4(b)) increased as time extended and liver function deteriorated in the control group. The caspase-1 and IL-18 expression in the BMSCs transplantation group was significantly lower than that in the control group, as shown in Figures 4(a) and 4(b). The caspase-1 and IL-18 protein expression in the BMSCs transplantation group decreased as liver function was improved. There were significant differences between the transplantation group and the control group at 120 h and 168 h after transplantation (*P* < 0.05). The correlation analysis showed that caspase-1 and IL-18 were both positively correlated with hepatocyte apoptosis (*P* < 0.01). These results suggest that BMSCs transplantation can reduce the expression of caspase-1 and IL-18 to balance the levels and functions of proinflammatory and anti-inflammatory cytokines. 

The real time PCR results (Figures 4(c) and 4(d)) indicated that caspase-1 and IL-18 mRNA expression was significantly lower in the BMSCs transplantation group than in the control group at 120 h and 168 h after transplantation (*P* < 0.05). In the BMSCs transplantation group, the caspase-1 and IL-18 mRNA expression was gradually decreased as liver function improved, and correlation analysis showed that caspase-1 and IL-18 were positively correlated (*rs* = 0.919, *P* < 0.01). These results suggest that BMSCs transplantation may affect caspase-1 and IL-18 expression at a transcriptional level. 

### 3.5. Expression of VEGF and AFP Protein

**Figure 5:**
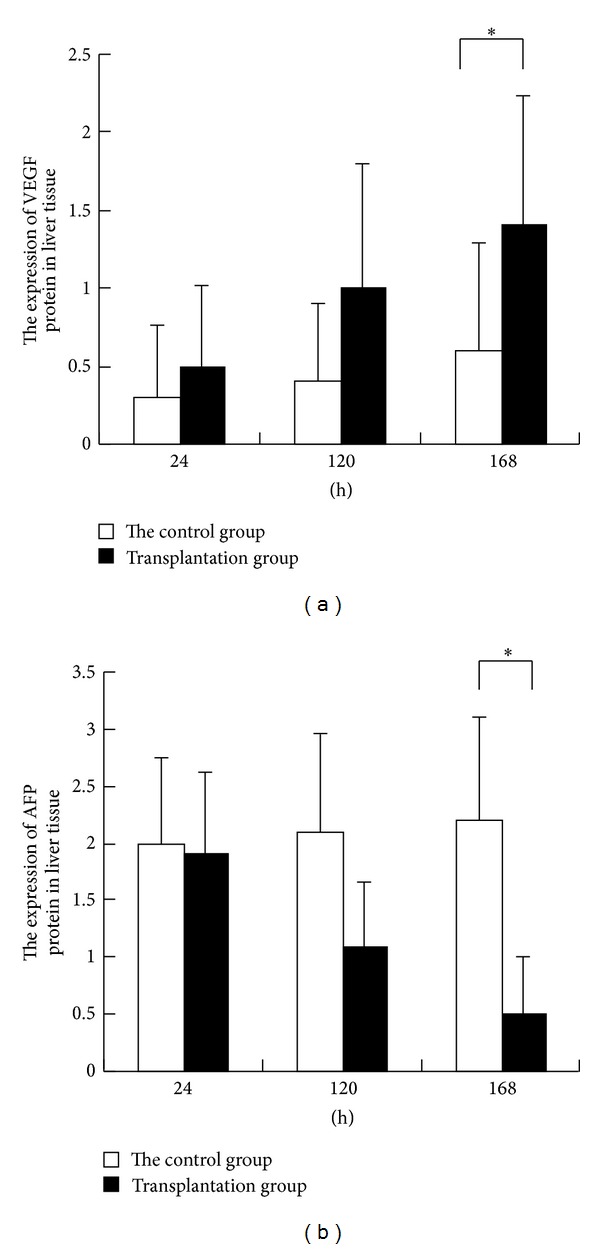
Immunofluorescence detection. The samples were prepared according to the protocol. VEGF antibody and the AFP antibody were used. FITC-labeled secondary antibody was added and the solution was incubated for 15 minutes. Then, DAPI was used to stain nucleus. The cells were observed under fluorescence microscope. The numbers of fluorescent-labeled liver cells and the total numbers of liver cells in each field were counted. (a) VEGF protein expression in liver tissues. (b) AFP protein expression in liver tissues.

To investigate the proliferation of liver cells by BMSCs transplantation, the immunofluorescence assay was performed to detect the expression of VEGF and AFP protein in liver tissue. The immunofluorescence results (Figure 5) showed that VEGF protein expression gradually increased with the improvement of liver function in the BMSCs transplantation group and the AFP protein expression gradually increased as time extended after BMSCs transplantation and the liver function deteriorated. There was a significant difference in the VEGF (Figure 5(a)) and AFP (Figure 5(b)) protein expression levels between the control group and the transplantation group (*P* < 0.05) at 168 h after transplantation. These results suggest that BMSCs may secrete VEGF to promote revascularization and liver cell proliferation.

## 4. Discussion

Studies have reported that BMSCs secrete cytokines and growth factors in a paracrine fashion to promote regeneration, inhibit inflammation, and reduce the generation of extracellular matrix and degradation of the intrahepatic excess in the extracellular matrix [12–14]. Studies have shown that BMSCs have the lasting ability to generate hepatocytes and bile duct cells in the repair process after liver injury when there is severe liver damage [15–17]. Therefore, BMSC therapy is currently a promising strategy in ALF treatment [18, 19]. 

In this study, D-galactosamine model/lipopolysaccharide-induced acute liver failure model was used. After establishment of the model, HE staining showed typical acute liver damage. Serum ALT and AST levels and liver cell apoptosis in control group were significantly increased, compared with those in transplantation group in a time-dependent manner, suggesting a successful modeling. At 120 h and 168 h after BMSCs transplantation, liver infiltration of inflammatory cells was significantly reduced, lobular was gradually restored, liver cell necrosis and apoptosis were significantly reduced compared with those of the control group, and rat liver function gradually improved. These results confirm that BMSCs transplantation can inhibit inflammatory response and liver cell apoptosis to repair the damaged liver tissue and BMSCs are of certain therapeutic effect for ALF rats. After liver cell transplantation, early immune rejection showed transplanted liver cell apoptosis, which is one of the important factors affecting the liver cell transplantation. Under normal circumstances, the liver cells will gradually go through apoptosis three days after transplantation, but acute liver failure patients need at least 5 to 7 days to recover liver function. Therefore, reduction of the occurrence of apoptosis in cell transplantation is the key to the effectiveness of cell transplantation.

IL-18 is a cytokine with a wide range of biological activity in various infectious diseases and autoimmune diseases [20–22]. Caspase-1 is a cysteine aspartate specific protease, which is an important member of the caspase family. Caspase-1 not only is involved in a variety of cell apoptosis but also plays roles in activation of the IL-18 [23–25]. By immunohistochemistry and RT-PCR, caspase-1 and IL-18 expression in liver tissue of ALF rats was detected. It was found that the expression trends were consistent, and with the aggravation of apoptosis, its expression gradually increased, indicating the role of caspase-1 and IL-18 in hepatocyte apoptosis. caspase-1 is an activating enzyme of IL-18, and the increase of these two proteins at the same time indicated that IL-18 in acute liver failure could be activated by Caspase-1 and thus played the role of induction of apoptosis. The results showed that with elevated serum ALT and AST levels, caspase-1 and IL-18 level was significantly increased, which was significantly higher than that in the transplantation group. The correlation of caspase-1, IL-18, and ALT, AST levels suggested the sharp deterioration of liver function and severity of disease and indicated the important role of caspase-1 and IL-18 in the pathogenesis of ALF. And caspase-1 and IL-18 can be used as sensitive marker protein for the diagnosis and prognosis of ALF. After treatment with stem cell transplantation, caspase-1 and IL-18 expression were decreased, and rat liver function gradually recovered, suggesting that BMSCs transplantation can improve the immunity of AHF rats and can be protective for ALF rats. One of the mechanisms of BMSC transplantation in the treatment of ALF is that it reduces caspase-1 and IL-18 expression, and thus it suppresses the immune cell proliferation and balances proinflammatory and anti-inflammatory cytokines. It is suggested that the trend of caspase-1 and IL-18 expression in the treatment can reflect the efficacy and prognosis of ALF to some extent; therefore, caspase-1 and IL-18 are expected to be predictors and future targets of acute liver failure.

VEGF is known to be the strongest growth factor for induction of angiogenesis. Okuyama et al. [26] found that in the transformation of BMSC to vascular endothelial-like cells, it first stimulated local microenvironment to promote VEGF receptor increase on BMSC surface. BMSC was transformed into vascular endothelial-like cells by local VEGF stimulation for the angiogenesis. It is found [27–29] that human BMSC promoted angiogenesis through paracrine mechanisms, and gene encoding angiogenesis related cytokines increased, such as VEGF, FGF-2, and IL-6. Parekkadan et al. [30] injected conditioned medium of bone marrow stem cell into mice and found a 90% reduction in liver apoptosis and a 3-time increase in regeneration. Liu et al. [31] reported that trauma can stimulate BMSC to produce a variety of cytokines, such as VEGF, FGF and HGF, to promote angiogenesis and damage repair. Therefore, paracrine signaling pathway is an important mediator in the bone marrow cell treatment for tissue ischemia.

It is found that after BMSC transplantation, liver function gradually improved, liver cell necrosis gradually reduced, and VEGF protein expression in liver tissue gradually increased. It is suggested that BMSCs secrete VEGF, promote hepatocyte proliferation and liver angiogenesis, and regulate immunity and inflammatory response of liver in hepatic failure rats.

The evaluation method of improved liver function includes the Child-Pugh and MELD score and blood biochemical parameters. AFP, PCNA (proliferating cell nuclear antigen), TNF, IL-6, and IL-10 were used to evaluate the degree of liver regeneration [32]. Robert Schwartz et al. [33] isolated human bone marrow hematopoietic stem cells to receive specific training and found that the expression of liver cell-specific surface markers of CK18, CK19, ALB, and AFP. This study showed that the level of AFP protein in liver tissue gradually increased with the deterioration of liver function and it reflected degrees of the liver cell proliferation to a different extent. At the same time, it provides an experimental basis for the evaluation of BMSCs transplantation for liver regeneration and functional recovery.

In this study, we investigated the role and mechanism of BMSC transplantation in rat model with ALF. BMSCs were injected through the tail vein. Through regulating immune responses in the liver, inhibiting liver cell apoptosis, and promoting liver angiogenesis and liver cell proliferation, BMSCs improved the liver function of ALF rats. Our study was different from the one reported by Li et al. [34]. They reported the effectiveness of BMSCs in the treatment of ALF using human BMSCs and pig ALF model. They also compared the effect of different transplantation route and observed hBMSC-derived hepatocytes. Our findings further confirmed the effectiveness of BMSCs in the treatment of ALF. However, the effect of different transplantation route and the percentage of BMSC-derived hepatocytes are not clear in this study, which needs further investigation.

In summary, our data suggest that BMSC transplantation is of therapeutic benefit for treating ALF by providing anti-inflammatory and antiapoptotic effects. Further studies are needed to clarify the exact mechanism and to improve the effectiveness of BMSCs transplantation.

## Figures and Tables

**Table 1:** Primers used in this study.

Primers	Sequences	Sizes of products
Caspase-1_F	5′-AACTGAACAAAGAAGGTGGCG-3′	142 bp
Caspase-1_R	5′-GCAAGACGTGTACGAGTGGGT-3′	

IL-18_F	5′-AACCGCAGTAATACGGAGCAT-3′	179 bp
IL-18_R	5′-CCTTCACAGATAGGGTCACAGC-3′	

*β*-Actin_F	5′-CGTAAAGACCTCTATGCCAACA-3′	229 bp
*β*-Actin_R	5′-CGGACTCATCGTACTCCTGCT-3′	
